# Connectivity Disruption, Atrophy, and Hypometabolism within Posterior Cingulate Networks in Alzheimer's Disease

**DOI:** 10.3389/fnins.2016.00582

**Published:** 2016-12-21

**Authors:** Justine Mutlu, Brigitte Landeau, Clémence Tomadesso, Robin de Flores, Florence Mézenge, Vincent de La Sayette, Francis Eustache, Gaël Chételat

**Affiliations:** ^1^Institut National de la Santé et de la Recherche Médicale, U1077Caen, France; ^2^Université de Caen Normandie UMR-S1077Caen, France; ^3^Ecole Pratique des Hautes Etudes, UMR-S1077Caen, France; ^4^CHU de Caen, U1077Caen, France; ^5^CHU de Caen, Service de NeurologieCaen, France

**Keywords:** posterior cingulate cortex, Alzheimer's disease, resting-state functional connectivity, atrophy, hypometabolism

## Abstract

The posterior cingulate cortex (PCC) is a critical brain network hub particularly sensitive to Alzheimer's disease (AD) and can be subdivided into ventral (vPCC) and dorsal (dPCC) regions. The aim of the present study was to highlight functional connectivity (FC) disruption, atrophy, and hypometabolism within the ventral and dorsal PCC networks in patients with amnestic mild cognitive impairment (aMCI) or AD. Forty-three healthy elders (HE) (68.7 ± 6 years), 34 aMCI (73.4 ± 6.8 years) and 24 AD (70.9 ± 9.1 years) patients underwent resting-state functional MRI, anatomical T1-weighted MRI and FDG-PET scans. We compared FC maps obtained from the vPCC and dPCC seeds in HE to identify the ventral and dorsal PCC networks. We then compared patients and HE on FC, gray matter volume and metabolism within each network. In HE, the ventral PCC network involved the hippocampus and posterior occipitotemporal and temporoparietal regions, whereas the dorsal PCC network included mainly frontal, middle temporal and temporoparietal areas. aMCI patients had impaired ventral network FC in the bilateral hippocampus, but dorsal network FC was preserved. In AD, the ventral network FC disruption had spread to the left parahippocampal and angular regions, while the dorsal network FC was also affected in the right middle temporal cortex. The ventral network was atrophied in the bilateral hippocampus in aMCI patients, and in the vPCC and angular regions as well in AD patients. The dorsal network was only atrophied in AD patients, in the dPCC, bilateral supramarginal and temporal regions. By contrast, hypometabolism was already present in both the vPCC and dPCC networks in aMCI patients, and further extended to include the whole networks in AD patients. The vPCC and dPCC connectivity networks were differentially sensitive to AD. Atrophy and FC disruption were only present in the vPCC network in aMCI patients, and extended to the dPCC network in AD patients, suggesting that the pathology spreads from the vPCC to the dPCC networks. By contrast, hypometabolism seemed to follow a different route, as it was present in both networks since the aMCI stage, possibly reflecting not only local disruption but also distant synaptic dysfunction.

## Introduction

Alzheimer's disease (AD) is the most widespread cause of dementia. This neurodegenerative disease is characterized by a progressive decline in cognitive performances, typically predominated by episodic memory deficits. Tau-rich neurofibrillary tangles and β-amyloid (Aβ) plaques are the two neuropathological landmarks of the disease. *In vivo* neuroimaging biomarkers of the disease include atrophy (predominantly in the hippocampus and temporal neocortex), hypometabolism (mainly in the posterior cingulate cortex (PCC), and temporo-parietal cortex), and amyloid deposition in medial frontal and parietal and temporo-parietal cortical areas (McKhann et al., 2011; Winblad et al., 2016). Moreover, connectivity has proved to be critical in the pathophysiology of the disease. Disconnection processes have been shown to be at least partly responsible for early hypometabolism in AD (Villain et al., 2010; Teipel and Grothe, 2016), and neuropathological research suggests that tau propagates transynaptically, neuron to neuron (Duyckaerts et al., 1997; Braak and Del Tredici, 2011; de Calignon et al., 2012; Ahmed et al., 2014). Finally, recent neuroimaging studies have shown that the topography of atrophy/hypometabolism in AD (and other forms of dementia) follows specific brain connectivity networks, as evidenced by resting-state functional magnetic resonance imaging (fMRI), for instance, leading to the network degeneration hypothesis (Seeley et al., 2009; La Joie et al., 2014). More specifically, the default mode network (DMN) includes those brain areas that are most sensitive to AD (i.e., sites of the earliest atrophy, hypometabolism and/or amyloid deposition), such as the PCC, precuneus, hippocampus, temporo-parietal, and medial frontal areas (Greicius et al., 2004; Sheline and Raichle, 2013).

Within this context, the PCC is thought to play a pivotal role as it is one of the main hubs of the DMN and connects different subsystems of this network (Buckner et al., 2008; Hagmann et al., 2008; Andrews-Hanna et al., 2010). Moreover, it is the earliest site of hypometabolism in AD (Minoshima et al., 1997; Chételat et al., 2008), which is thought to reflect disconnection from the hippocampus (Villain et al., 2008, 2010; Yakushev et al., 2011) and, in turn, to be related to hypometabolism in distant brain regions such as the frontal cortex (Fouquet et al., 2009; Villain et al., 2010). Finally, the PCC is involved in episodic/autobiographical memory processes (Cabeza and Nyberg, 2000; Maddock et al., 2001; Wagner et al., 2005; Svoboda et al., 2006; Fossati, 2013; Andrews-Hanna et al., 2014), and disruption of PCC connectivity and metabolism is known to be at least partly responsible for early episodic memory deficits in AD and amnestic mild cognitive impairment (aMCI) (Chételat et al., 2003; Bastin et al., 2010; La Joie et al., 2014).

The PCC is a heterogeneous structure, with cytoarchitectonic differences along the dorso-ventral axis (Vogt, 2005; Vogt et al., 2006) and in the receptor architecture (Palomero-Gallagher et al., 2009). Previous neuroimaging studies using a variety of techniques (e.g., diffusion tensor imaging, resting-state glucose metabolism, and resting-state fMRI) in healthy individuals have also highlighted specificities in the functional connectivity (FC) of the ventral (vPCC) vs. dorsal (dPCC) PCC. Thus, the vPCC seems to be more strongly connected to medial temporal areas (Greicius et al., 2004; Vogt et al., 2006), including the hippocampus and parahippocampal gyrus (Maddock et al., 2001; Beckmann et al., 2009; Margulies et al., 2009), orbitofrontal and ventromedial prefrontal cortex (Vogt et al., 2006; Bzdok et al., 2015), anterior cingulate (Maddock et al., 2001; Vogt et al., 2006), occipital cortex (Vogt et al., 2006) and left inferior part of the parietal cortex (Bzdok et al., 2015). By contrast, the dPCC appears to be connected to the prefrontal cortex, especially its dorsomedial (Beckmann et al., 2009; Bzdok et al., 2015), dorsolateral (Bzdok et al., 2015) and ventromedial parts (Greicius et al., 2009; Margulies et al., 2009), the parietal cortex (Vogt et al., 2006; Beckmann et al., 2009) and the lateral temporal cortex (Margulies et al., 2009). Thus, while both parts of the DMN (Yu et al., 2011), each subregion yet belongs to distinct subnetworks.

Given the pivotal role of the PCC in AD and within the DMN, and the distinct cellular organization and connectivity profile of the vPPC vs. dPCC, the goal of the present study was to investigate structural and functional alterations of the vPPC and dPCC networks in patients with aMCI and demented patients with AD. More specifically, we sought to (i) identify the specific connectivity subnetworks of the vPCC vs. the dPCC in cognitively normal older adults and (ii) assess the profiles of connectivity disruption, atrophy and hypometabolism within the vPCC and dPCC networks in patients with aMCI or AD.

## Materials and methods

### Participants

One hundred and one right-handed native French-speaking participants from the ≪ Imagerie Multimodale de la maladie d'Alzheimer à un stade Précoce ≫ (IMAP+) study (Caen) were included in the present study: 43 healthy elders (HE), 34 patients with aMCI, and 24 patients with AD (Table 1). Some of them had been included in previous publications by our laboratory (La Joie et al., 2012, 2013, 2014; Mevel et al., 2013; Tomadesso et al., 2015). All participants were aged over 60 years, had at least 7 years of education, and had no history of alcoholism, drug abuse, head trauma, or psychiatric disorder.

**Table 1:** **Demographic information of healthy elders (HE), patients with amnestic mild cognitive impairment (aMCI), and patients with Alzheimer's disease (AD)**.

	**HE**	**aMCI**	**AD**	***p*-value**			
				**Group effect**	**HE vs. aMCI**	**HE vs. AD**	**aMCI vs. AD**
F/M ratio	27/16	18/16	12/12	–	0.523	0.448	0.963
Age in years (*SD*)	68.7 (6.0)	73.4 (6.8)	70.5 (9.4)	**0.020**	**0.022**	0.532	0.460
Education in years (*SD*)	12 (3.5)	10 (3.5)	10 (3.3)	0.116	0.192	0.319	0.999
MMSE (*SD*)	29.2 (0.8)	26.7 (1.7)	20.8 (4.2)	***p* < 0.001**	***p* < 0.001**	***p* < 0.001**	***p* < 0.001**

*The bold values are significant (p < 0.05)*.

HE were recruited from the community and performed within the normal range on all neuropsychological tests in a cognitive battery assessing multiple domains of cognition (verbal and visual episodic memory, semantic memory, language skills, executive functions, visuospatial functions, and praxis). The patients with aMCI or AD were recruited from local memory clinics and selected according to internationally agreed criteria. aMCI patients were selected based on Petersen's criteria for aMCI (Petersen and Morris, 2005) and AD patients fulfilled standard National Institute of Neurological and Communicative Disorders and Stroke, and Alzheimer's Disease and Related Disorders Association (NINCDS-ADRDA) clinical criteria for probable Alzheimer's disease (McKhann et al., 1984). Clinical diagnosis was assigned by consensus under the supervision of a senior neurologist (VdLS) and two neuropsychologists (AP and SE). The majority of participants underwent a florbetapir-PET scan, and the proportions of amyloid-positive scans using previously published methods (La Joie et al., 2012) were 17.9% (7/39) for HE, 65.6% (21/32) for patients with aMCI, and 100% (23/23) for patients with AD.

The IMAP+ study was approved by a regional ethics committee (Comité de Protection des Personnes Nord-Ouest III) and registered with http://clinicaltrials.gov (no. NCT01638949). All participants gave their written informed consent to the study prior to the investigation.

### Neuroimaging data acquisition

All participants were scanned with the same MRI and PET cameras at the Cyceron Center (Caen, France): a Philips Achieva 3.0 T scanner and a Discovery RX VCT 64 PET-CT device (General Electric Healthcare), respectively. High-resolution T1-weighted anatomical volumes were acquired using a three-dimensional fast-field echo sequence (3D-T1-FFE sagittal; repetition time = 20 ms, echo time = 4.6 ms, flip angle = 10°, 180 slices with no gap, slice thickness = 1 mm, field of view = 256 × 256 mm^2^, in-plane resolution = 1 × 1 mm^2^).

Resting-state functional volumes were obtained using an interleaved 2D T2^*^ SENSE EPI sequence designed to reduce geometric distortions, with parallel imaging, short echo time, and small voxels (2D-T2^*^-FFE-EPI axial, SENSE = 2; time repetition = 2382 ms, time echo = 30 ms, flip angle = 80°, 42 slices with no gap, slice thickness = 2.8 mm, field of view = 224 × 224 mm^2^, in plane resolution = 2.8 × 2.8 mm^2^, 280 volumes, acquisition time = 11.5 min). Participants were equipped with earplugs, their head was stabilized with foam pads to minimize head motion, and the scanner room's light was turned off. During this acquisition, which was the last one in the MRI scanning session, participants were asked to relax, lie still in the scanner, and keep their eyes closed, without falling asleep. A subsequent debriefing questionnaire allowed us to ensure that the participants had no difficulty staying awake throughout the duration of the resting-state fMRI scan and that nothing particular had disturbed their attention during the scanning.

Finally, ^18^FDG-PET images were acquired with a resolution of 3.76 × 3.76 × 4.9 mm^3^ (field of view = 157 mm). Forty-seven planes were obtained with a voxel size of 1.95 × 1.95 × 3.2 mm^3^. A transmission scan was performed for attenuation correction before the PET acquisition. Participants were fasted for at least 6 h before scanning. After a 30-min resting period in a quiet and dark environment, 180 MBq of FDG was intravenously injected as a bolus. A 10-min PET acquisition scan began 50 min after injection.

### Neuroimaging data preprocessing

#### Anatomical MRI

MRI data were segmented, normalized to the Montreal Neurological Institute (MNI) template and modulated (nonlinear only) using the VBM5.1 toolbox, and smoothed with an 8-mm Gaussian filter. The resulting gray matter datasets were used in all subsequent analyses.

#### Resting-state fMRI

Individual datasets were first checked for artifacts by applying the TSDiffAna routines (http://imaging.mrc-cbu.cam.ac.uk/imaging/DataDiagnostics), during which a variance volume was created for each participant to check that most of the signal variability was restricted to the cortex. Datasets showing evidence of significant movements (>3 mm translation or 1.5° rotation) associated with image artifacts and/or an abnormal variance distribution were excluded from subsequent analyses. Data were then processed as described in La Joie et al. (2014), including slice timing correction, realignment to the first volume, spatial normalization, smoothing (4 mm), masking to include only gray matter voxels and exclude the cerebellum (based on the T1-weighted and non-EPI-T2^*^ volumes), and temporal bandpass filtering (0.01–0.08 Hz).

The vPCC and dPCC were manually delineated on the normalized anatomical T1-MRI of a representative HE scan using Anatomist (Version 4.0.0) software, based on cytoarchitectural and functional findings (Vogt et al., 2006; Yu et al., 2011). The vPCC included Brodmann areas v23 and 31, and was bordered anteriorly by the ventral branch of the splenial sulcus and posteriorly by the parieto-occipital sulcus. The dPCC included Brodmann areas 23c, 23d, d23, and 31, and was limited anteriorly by the cingulate sulcus and posteriorly by the ventral branch of the splenial sulcus.

**Figure 1:**
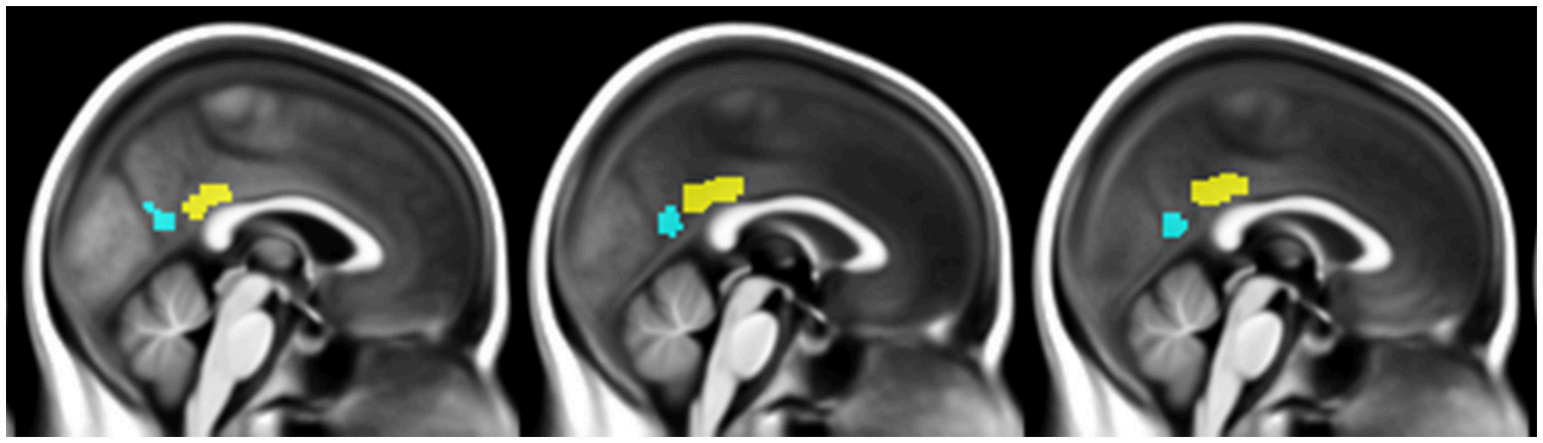
**Illustration of the ventral PCC (blue) and dorsal PCC (yellow) seeds manually delineated on the mean normalized anatomical T1-MRI scans of all participants**.

The two subregions were then masked using the gray matter mask described above, and used as seeds in subsequent connectivity analyses (Figure 1). Coincidently, both regions measured 3592 mm^3^ (449 voxels). We then checked that the vPCC and dPCC locations matched for each participant, by superimposing the regions on each individual normalized scan.

For each of the 101 participants and each seed of interest (transformed using the MarsBar toolbox, Brett et al., 2002), positive correlations were assessed between the mean time course in the seed and the time course of each gray matter voxel. To remove potential sources of spurious variance, the time courses from white matter, cerebrospinal fluid, the whole brain, their derivatives, and the six movements parameters generated from realignment of head motion were introduced as covariates. Lastly, Fisher's z transform and 6.3 mm full width at half maximum (FWHM) smoothing were applied to the resulting individual connectivity maps, leading to a final smoothness of 8 mm FWHM (√(2.8^2^ + 4^2^ + 6.3^2^)).

#### FDG-PET

^18^FDG-PET data were corrected for partial volume effects using a three-compartment method (Giovacchini et al., 2004) with PMOD (PMOD Technologies). Images were then coregistered onto their respective MRIs, spatially normalized to the MNI template using the respective MRI parameters, and scaled using the mean PET value of the cerebellar gray matter. Smoothing kernel of 7.1 × 7.1 × 6.3 mm Gaussian filter was applied so that the final smoothness of the images was the same as for the fMRI data (8 × 8 × 8 mm).

### Statistical analysis

Between-group differences on demographic variables were assessed with a one-factor (group) analysis of variance (ANOVA) for continuous variables and a chi2 test for categorical variables.

#### Identification of the vPCC and dPCC networks in HE

**Figure 2:**
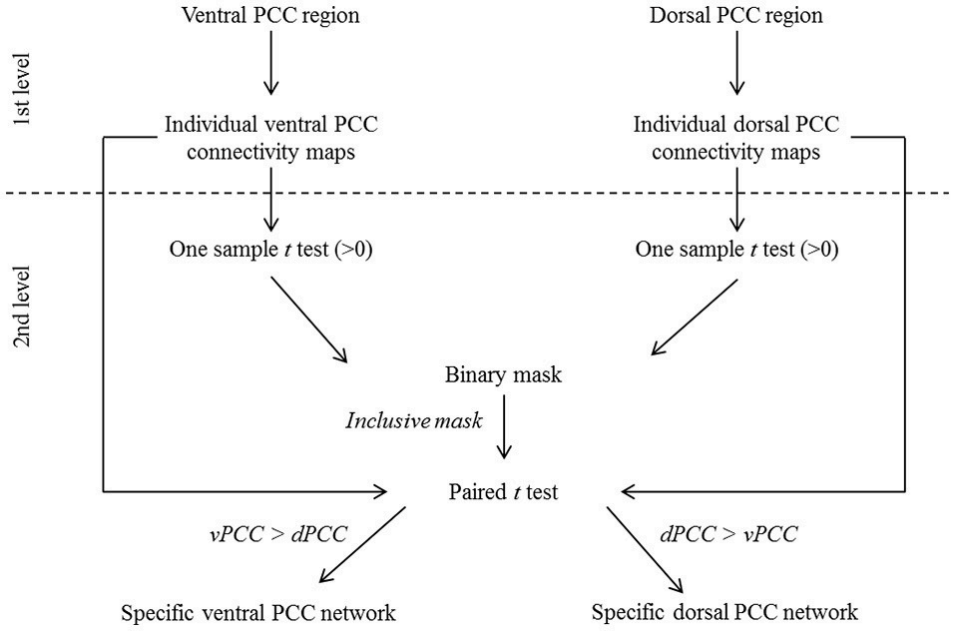
**Schematic representation of the procedure for ventral (vPCC) and dorsal (dPCC) PCC connectivity analyses in HE**. At the first level, vPCC and dPCC regions were used as seeds to obtain individual vPCC and dPCC connectivity maps. At the second level, one-sample *t*-tests (>0) were performed on the individual connectivity maps and thresholded at *p* (FWE-corrected) <0.01, k > 100 to identify regions positively correlated with the vPCC and dPCC in HE. The resulting maps were binarized and combined to obtain a mask of brain regions that were positively correlated with the ventral or dorsal PCC. This mask was used in a paired *t*-test comparing the individual vPCC and dPCC connectivity maps to identify, within the regions positively associated with the ventral or dorsal PCC, those more correlated with one or the other. The resulting maps, thresholded at *p* (FWE-corrected) < 0.05 (k > 50) and binarized, were used as the specific ventral and dorsal PCC networks in subsequent analyses.

**Figure 3:**
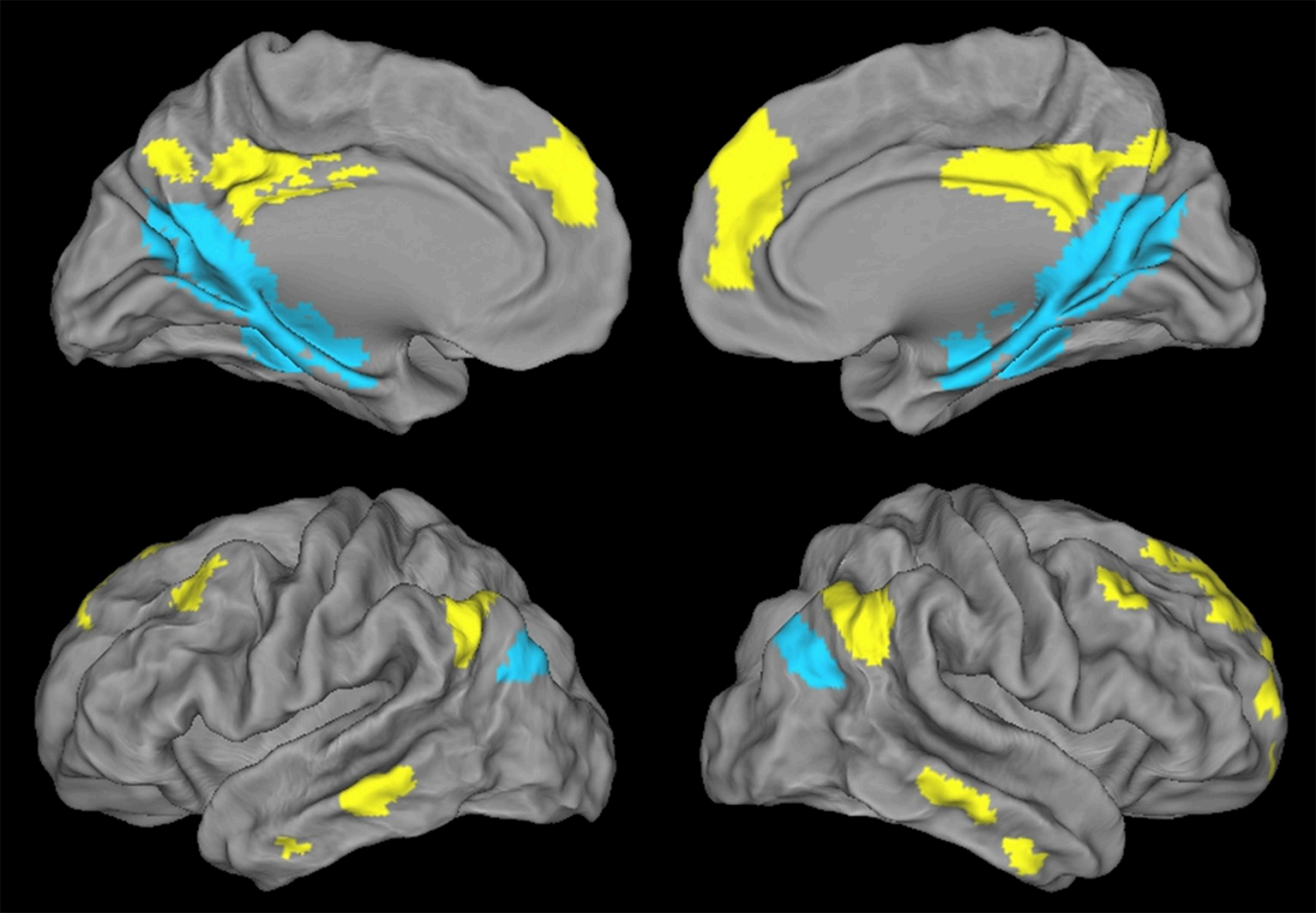
**Illustration of the ventral PCC (blue) and dorsal PCC (yellow) networks in HE obtained with a paired *t*-test and thresholded at *p* (FWE-corrected) < 0.05, k > 50**.

A voxelwise paired *t*-test was performed on resting-state fMRI scans in HE to identify the brain regions that were significantly more correlated with the dPCC than with the vPCC and vice versa (FWE-corrected *p* < 0.05, k > 50), including only regions that were significantly positively correlated with these regions in HE (see details in Figure 2). Binary masks of the ventral and dorsal networks were obtained from this analysis for use in subsequent analyses (Figure 3).

#### Voxelwise neuroimaging analyses in patients

To assess changes within these networks in patients with aMCI or AD, compared with HE, we ran single factor (group) ANCOVAs using Statistical Parametric Mapping (SPM) software with full factorial designs. More specifically, two ANCOVAs were carried out to assess FC disruption in patients vs. HE within the ventral and dorsal networks, and the same analyses were repeated with maps of gray matter volume and ^18^FDG-PET metabolism to assess for atrophy and hypometabolism within these same networks. Age, sex, and education were controlled for in all group comparisons, and we used a statistical threshold of *p* (uncorrected) = 0.001. Cluster extent was determined by Monte-Carlo simulation (AlphaSim program by D. Ward) for each modality and within each network to achieve a multiple comparison-corrected *p* < 0.05 (Table S1). The percentage of alteration within each network was calculated by dividing the total number of disrupted voxels by the total number of voxels in the corresponding network (Table 2). Finally, to assess the links with global cognitive changes, correlations were assessed between atrophy and hypometabolism found in vPCC and dPCC networks in aMCI and AD patients vs. performances at the MMSE using non-parametric Spearman correlation analyses and a *p* < 0.05 (Figure S2).

**Table 2:** **Percentage of the volume of the ventral and dorsal networks showing FC disruption, atrophy and hypometabolism in patients compared with HE**.

	**Ventral Network**		**Dorsal Network**	
	**MCI**	**AD**	**MCI**	**AD**
FC disruption	1.82	3.17	0.00	0.47
Atrophy	11.42	49.37	0.00	50.23
Hypometabolism	10.09	61.21	28.18	79.24

## Results

### Demographic data

The three groups did not differ on either sex or years of education, but the patients with aMCI were significantly older than HE (Table 1).

### Identification of the vPCC and dPCC networks in HE

Regions showing higher connectivity with the vPCC than with the dPCC, and thus constituting the ventral PCC network, included the vPCC region (autocorrelation) extending to the retrospenial cortex, the medial temporal lobe including the hippocampus and parahippocampal cortex and encroaching on the fusiform and lingual gyri, the ventroposterior part of the precuneus, the cuneus, the most inferoposterior part of the angular gyrus, and the parieto-occipital sulcus (Figure 3). The volume of the vPCC network was 53272 mm^3^ (6659 voxels).

Regions showing higher connectivity with the dPCC than with the vPCC, and thus constituting the dorsal PCC network, bilaterally included the dPCC region (autocorrelation) extending to the middle cingulate cortex, the dorsomedial prefrontal cortex (encroaching on both the anterior cingulate gyrus and the frontal superior cortex), and the superior frontal gyrus (superolateral part), middle frontal gyrus (superior portion), orbitofrontal cortex, dorsal part of the precuneus, supero-anterior part of the angular gyrus, and middle temporal gyrus (Figure 3). The volume of the dPCC network was 58,968 mm^3^ (7371 voxels).

We had expected to highlight distinct FC networks for the ventral and dorsal PCC, consistent with previous studies and with the known heterogeneity of the PCC. We did indeed find significant differences between the two networks in HE, with the vPCC being more strongly connected to medial temporal and parieto-occipital brain areas, while the dPCC network mainly involved frontal and lateral temporal brain regions.

### Alteration within the vPCC and dPCC networks in patients with aMCI or AD, compared with HE

#### FC disruptions

Within the vPCC network, FC was bilaterally reduced in the anterior part of the hippocampus in patients with aMCI compared with HE. Within the dPCC network, no significant difference in FC was found between patients with aMCI and HE.

**Figure 4:**
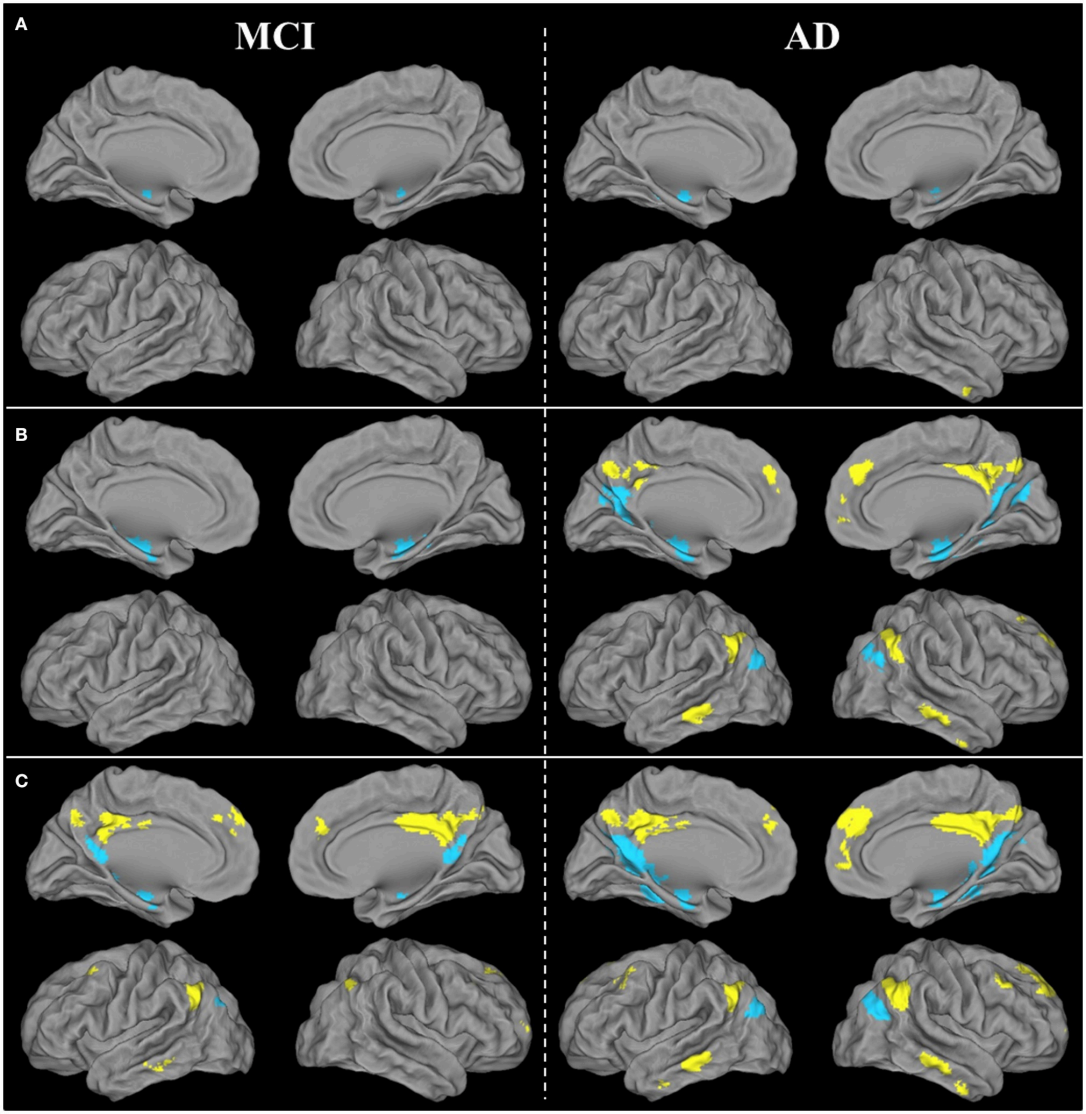
**Brain areas showing significant FC disruptions (A)**, atrophy **(B)**, and hypometabolism **(C)** within the ventral (blue) and dorsal (yellow) PCC networks of patients with aMCI or AD compared with HE, as revealed by ANCOVAs thresholded at *p* (uncorrected) <0.001.

In AD, FC disruptions within the vPCC network were found in the anterior part of the bilateral hippocampus, as well as in the left parahippocampal gyrus and left angular gyrus (inferoposterior part). Within the dPCC network, FC was reduced in the anterior part of the right middle temporal gyrus (Figure 4A).

Results therefore showed that FC disruption initially concerned the vPCC network at the aMCI stage. It then extended further within this network and also occurred within the dPCC network at the AD dementia stage.

#### Atrophy

Within the vPCC network, significant atrophy was found in the bilateral hippocampus and parahippocampal gyrus in patients with aMCI compared with HE. Within the dPCC, no significant atrophy was found in patients with aMCI vs. HE.

In patients with AD, the vPCC network was atrophied in the bilateral hippocampus, parahippocampal and fusiform gyri, bilateral vPCC, cuneus and posterior part of the precuneus, angular gyrus, and right parieto-occipital sulcus. The dPCC network was also atrophied bilaterally in patients with AD vs. HE in the supero-anterior part of the angular gyrus, dPCC and precuneus, middle temporal gyrus, anterior cingulate gyrus extending to the superior frontal gyrus, and in the right middle frontal gyrus (Figure 4B).

In sum, as with FC disruption, atrophy was initially restricted to the vPCC network at the aMCI stage, before extending further within this network and in the dPCC network as well at the AD dementia stage.

#### Hypometabolism

Within the vPCC network, significant bilateral hypometabolism was found in the vPCC/precuneus region and hippocampus in patients with aMCI compared with HE. The dPCC network was also hypometabolic in patients with aMCI, in parietal regions encompassing the bilateral dPCC and left angular cortex, as well as in the left inferoposterior temporal, bilateral orbitofrontal and middle frontal cortex.

In patients with AD vs. HE, significant hypometabolism was found in all the regions of the vPCC and dPCC networks (Figure 4C).

Thus, unlike FC disruption and atrophy, hypometabolism was found not to be restricted to the vPCC network at the MCI stage, as it was already present in both networks, and affected the whole of the vPCC and dPCC networks by the AD dementia stage.

#### Correlations between atrophy or hypometabolism and MMSE score

Atrophy and hypometabolism in MCI and AD in the vPCC and dPCC networks was found to be correlated with the MMSE score either significantly or at a trend level (rho values between 0.3 and 0.51 and *p*-values between 0.09 and 0.01; see Table S2 and Figure S2). This suggests that the alterations reflect the evolution of the pathological process associated with global cognitive decline.

## Discussion

In the present study, we sought to highlight the distinct FC of the vPCC and dPCC networks in HE, and identify the FC disruptions, atrophy, and hypometabolism within these two networks in patients with aMCI or AD.

### Topography of vPCC and dPCC networks in HE

Direct comparisons between vPCC vs. dPCC connectivity revealed the distinct topography of these networks in HE. The vPCC network mainly included parieto-occipital and medial temporal regions, while the dPCC network was mainly composed of frontal and lateral temporal brain regions. This topography is consistent with previous resting-state FC studies showing that the vPCC is more correlated with hippocampal and parahippocampal networks (Margulies et al., 2009) and the left inferior parietal cortex (Bzdok et al., 2015), while the dPCC is less correlated with the medial temporal lobe but more with the lateral temporal cortex (Margulies et al., 2009) and dorsomedial prefrontal, inferior parietal, posterior middle cingulate and left dorsolateral prefrontal cortex (Bzdok et al., 2015). These findings were also corroborated by a correlation analysis of resting-state glucose metabolism data showing higher correlations for the vPCC with the temporal and occipital cortices, and higher correlations for the dPCC with parietal areas (Vogt et al., 2006). Despite this overall consistency, there were also subtle differences from previous studies. For example, the prefrontal cortex was described as forming part of the vPCC network in some previous studies (Maddock et al., 2001; Bzdok et al., 2015) but not here, or the parietal cortex has sometimes been found to belong to the dPCC network (Vogt et al., 2006; Beckmann et al., 2009; Bzdok et al., 2015). These discrepancies probably reflect differences in the specific seed of interest, the samples, or other methodological aspects across studies. In line with their cytoarchitectonic and connectivity differences, distinct cognitive roles have been attributed to the ventral and dorsal PCC. Thus, the vPCC has been preferentially associated with tasks involving internally-focused attentional states such as self-reflection (Johnson et al., 2002), episodic (autobiographical) memory retrieval (Nielsen et al., 2005; Sugiura et al., 2005; Vogt et al., 2006; Dastjerdi et al., 2011), planning for the future (Fransson, 2005) and daydreaming (Mason et al., 2007). By contrast, the dPCC has been found to be preferentially involved in more externally-oriented attentional and/or visuospatial tasks (Maguire et al., 1997, 1998; Sugiura et al., 2005; Spreng and Schacter, 2012). The dPCC has also been found to play a role in switching between the DMN (internal focus) and the cognitive control network (external attention; Leech et al., 2011).

### Earlier atrophy and functional disruption in the vPCC network

The vPCC network showed alteration in both FC and gray matter volume, mainly in bilateral hippocampal regions, in patients with aMCI, whereas the dPCC network showed neither FC disruption nor atrophy at the aMCI stage. This early disruption of the vPCC network especially involving the hippocampus, is consistent with previous findings highlighting the particular vulnerability of the PCC-hippocampus axis in the course of AD (Villain et al., 2008, 2010), and studies showing alteration of the caudal part of the cingulum bundle that connects the hippocampus to the PCC (Choo et al., 2010; Villain et al., 2010). This vPCC network alteration may reflect the propagation of tau lesions from early sites of tau deposition, such as the medial temporal areas (Buée and Delacourte, 2006), to the most directly connected brain regions (vPCC network). It may also reflect the fact that the vPCC contains more pyramidal neurons than the dPCC (Vogt et al., 2006), and pyramidal neurons of the CA1 (Braak and Braak, 1997) are particularly vulnerable to tau pathology. Neuropathological examinations in patients with AD have also shown that the IV, Va, and Vb layers of the PCC (Broadmann area 23a) are the most vulnerable to neuronal loss (Vogt et al., 1998). These layers are more extensively represented and contain more densely packed neurons in the vPCC than in the dPCC (Vogt et al., 2006), which may explain the earlier involvement of the vPCC network in the present study.

By the AD dementia stage, the FC disruption and atrophy had extended further within the vPCC network (left fusiform and angular region), and was also present in the dPCC network (right middle temporal region for FC, and bilateral precuneus, frontal and temporal regions for atrophy). These results are consistent with the hypothesis that lesions propagate from the hippocampal-PCC axis within the vPCC network to the dPCC network, resulting in both FC disruption and atrophy, conducting to dementia (see below).

### The PCC as a hub through which the pathology spreads?

Medial temporal atrophy is one of the earliest events in the pathophysiological process of AD, and is thought to reflect neuronal and synaptic loss related to local neurofibrillary tangles (Fukutani et al., 2000; Whitwell et al., 2012). Our findings are consistent with the idea that the pathology (reflected in structural and FC disruptions), is initially restricted to the vPCC network, and subsequently spreads to the dPCC network. The PCC thus appears as a hub through which the pathology progresses. The two PCC subregions are known to be highly interconnected in monkeys (Parvizi et al., 2006) and humans (Leech et al., 2011). This hypothesis is in line with the network degeneration hypothesis whereby neurodegenerative diseases target brain networks (Seeley et al., 2009; Zhou et al., 2013; Raj et al., 2015, 2012) as pathologies progress from neuron to neuron through transneural spread, consistent with *prion-like* spreading (Frost and Diamond, 2009). Thus, misfolded proteins may spread throughout specific neural networks via trans-synaptic transmission pathways. Within this framework, we can hypothesize that the alterations observed within the vPCC network at the aMCI stage spread to the dPCC network in AD via the trans-synaptic connections between the vPCC and the dPCC.

### Hypometabolism within vPCC and dPCC

In contrast to the atrophy and connectivity disruptions, hypometabolism was found in both the vPCC and dPCC networks, even in patients with aMCI. Post-mortem studies in patients with AD suggest that PCC hypometabolism may, at least partly, be related to a reduction in the expression of energy metabolism genes in PCC neurons (Liang et al., 2008). Hypometabolism seems not to follow the same progressive involvement of the ventral and dorsal networks, as its progression was not initially restricted to the vPCC network. This may reflect the fact that hypometabolism is due to both local and distant alterations (La Joie et al., 2012), that is, diaschisis due to atrophy in connected brain regions (Chételat et al., 2009). In other words, atrophy within structures of the vPCC network in patients with aMCI may be associated with hypometabolism both in the same regions of the vPCC network and in distant connected areas of the dPCC network.

At the AD dementia stage, the whole vPCC and dPCC networks were hypometabolic. Early hypometabolism is commonly found in the PCC, temporoparietal and frontal areas in patients with AD or aMCI, and is thought to reflect mechanisms of disconnection from the atrophied hippocampus via the cingulum bundle and uncinate fasciculus (Chételat et al., 2008; Villain et al., 2008, 2010; Fouquet et al., 2009; Yakushev et al., 2011).

## Limitations/perspectives

Our study had several strengths and also several limitations. The strengths included a voxelwise multimodal approach that afforded a comprehensive view of the patterns of FC disruption, atrophy and hypometabolism in the vPCC and dPCC networks in patients with aMCI or AD. The limitations included the heterogeneous amyloid status of the patients with aMCI. However, when we repeated our neuroimaging analysis considering just the subgroup of amyloid-positive patients with aMCI (*n* = 21), the results remained essentially unchanged (Figure S1). The only difference was that, while there was no atrophy in the dPCC network for the aMCI group as a whole, atrophy was found in a small cluster located in the dPCC when we only considered the amyloid-positive patients with aMCI. The cross-sectional nature of the present study was a second limitation; further longitudinal studies would allow to demonstrate the spread of lesions from the vPCC to the dPCC networks and to assess the sequence of events and causal relationships between the different biomarkers.

## Ethics statement

Comite de Protection des Personnes Nord-Ouest III, registered with http://clinicaltrials.gov (number NCT01638949). All participants gave written informed consent to the study prior to the investigation. Alzheimer patients were accompanied by a relative who gave also his consent to the study.

## Author contributions

JM, GC, VdLS, and FE contributed to the study concept and design. JM, GC contributed to the interpretation of the data and to the drafting of the manuscript. JM, CT, RdF, and FM contributed to the data acquisition. JM, BL, and FM contributed to the data analyses. All the authors revised the work, approved the manuscript to be published, and agreed to be accountable for the work.

## Funding

This work was supported by the Programme Hospitalier de Recherche Clinique (PHRC National 2011 and 2012), the Agence Nationale de la Recherche (ANR LONGVIE 2007), the Région Basse Normandie and the Institut National de la Santé et de la Recherche Médicale (INSERM).

### Conflict of interest statement

The authors declare that the research was conducted in the absence of any commercial or financial relationships that could be construed as a potential conflict of interest.
