# Supplementary material for: Connectivity Disruption, Atrophy, and Hypometabolism within Posterior Cingulate Networks in Alzheimer's Disease
Source: Front Neurosci. 2016 Dec 21;10:582. doi: 10.3389/fnins.2016.00582 (PMC5174151; doi:10.3389/fnins.2016.00582)
Supplement: Supplementary file 1 [file Table1.DOCX]

Supplementary Material

Connectivity disruption, atrophy and hypometabolism within posterior cingulate networks in Alzheimer’s disease

Justine Mutlu ^1,2,3,4^, Brigitte Landeau ^1,2,3,4^, Clémence Tomadesso ^1,2,3,4^, Robin de Flores ^1,2,3,4^, Florence Mézenge ^1,2,3,4^, Vincent de La Sayette ^1,2,3,5^, Francis Eustache ^1,2,3,4^, Gaël Chételat ^1,2,3,4,*^

^1^ INSERM, U1077, Caen, France

^2^Université de Caen Basse-Normandie UMR-S1077, Caen, France

^3^Ecole Pratique des Hautes Etudes, UMR-S1077, Caen, France

^4^CHU de Caen, U1077, Caen, France

^5^CHU de Caen, Service de Neurologie, Caen, France

***Correspondence:** Gaël Chételat: [chetelat@cyceron.fr](mailto:chetelat@cyceron.fr)

Table S1: Cluster extent for each modality and within each network to achieve a multiple comparison-corrected statistical significance of *p* < 0.05.

|  | **Ventral Network** | | **Dorsal Network** | |
| --- | --- | --- | --- | --- |
|  | k | volume (mm^3^) | k | volume (mm^3^) |
| **FC** | 22 | 176 | 20 | 160 |
| **Gray Matter Volume** | 276 | 276 | 174 | 174 |
| **Metabolism** | 22 | 176 | 20 | 160 |

Table S**2**: Spearman correlations between atrophy or hypometabolism within vPCC and dPCC networks and MMSE score in patients with aMCI or AD with a significant threshold (*) set to *p* < 0.05.

|  |  | vPCC network | | dPCC network | |
| --- | --- | --- | --- | --- | --- |
|  |  | *rho* | *p* | *rho* | *p* |
| aMCI patients | Atrophy | 0.39 | 0.02* | - | - |
|  | Hypometabolism | 0.30 | 0.09 | 0.36 | 0.04* |
| AD patients | Atrophy | 0.36 | 0.09 | 0.51 | 0.01* |
|  | Hypometabolism | 0.47 | 0.03* | 0.46 | 0.04* |
