# Supplementary figures and images for: Connectivity Disruption, Atrophy, and Hypometabolism within Posterior Cingulate Networks in Alzheimer's Disease
Source: Front Neurosci. 2016 Dec 21;10:582. doi: 10.3389/fnins.2016.00582 (PMC5174151; doi:10.3389/fnins.2016.00582)

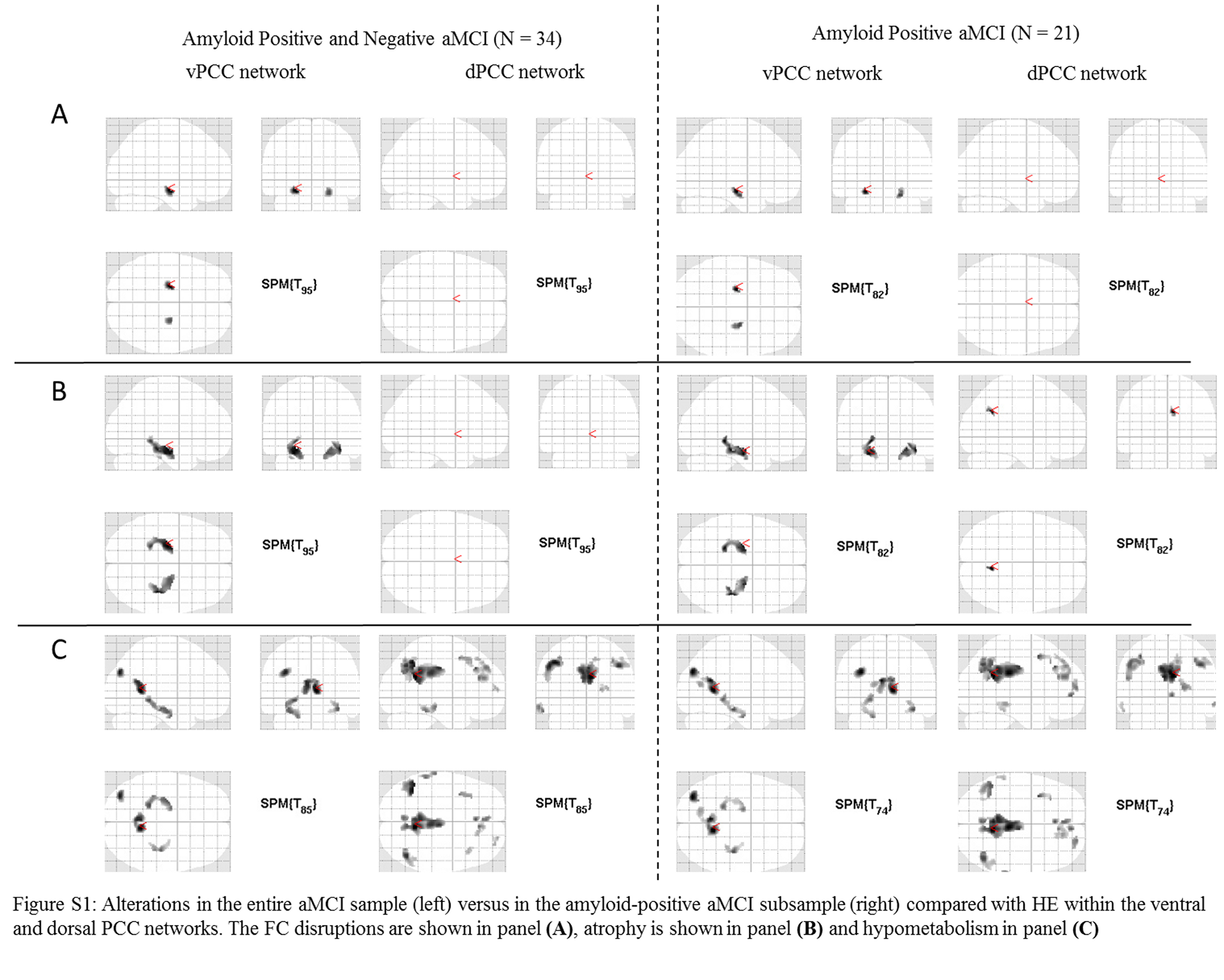

Supplement: Supplementary file 2 [file Image1.TIF]

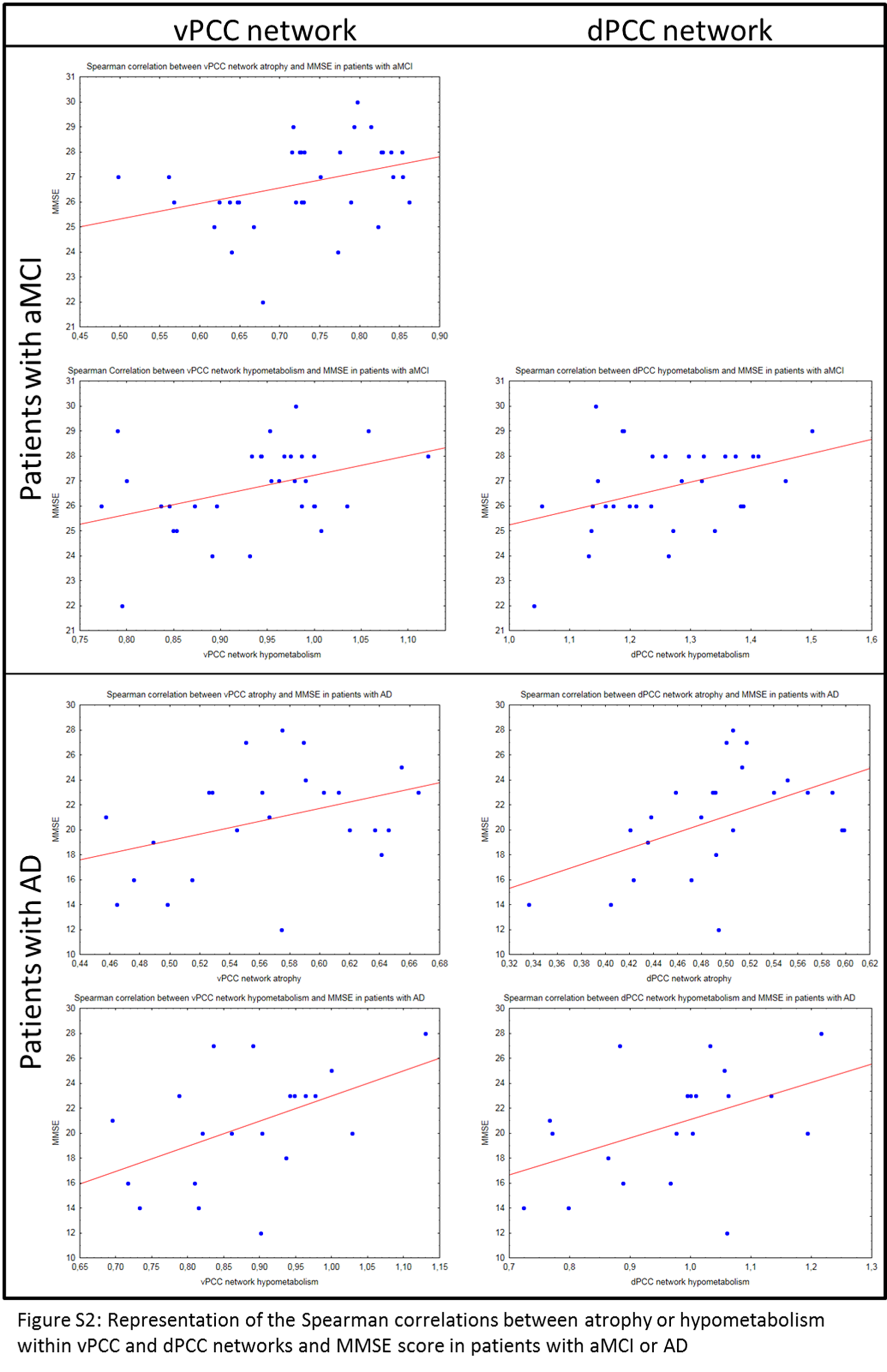

Supplement: Supplementary file 3 [file Image2.TIF]
